# New Technologies for Personalized Medicine in Head and Neck Oncologic and Reconstructive Surgery

**DOI:** 10.3390/jcm11154261

**Published:** 2022-07-22

**Authors:** José Luis Cebrián Carretero, Carlos Navarro Cuéllar

**Affiliations:** 1 Department of Oral and Maxilofacial Surgery, Hospital Universitario La Paz, Universidad Autónoma de Madrid, IDIPAZ, 28046 Madrid, Spain; 2 División of Oral and Maxillofacial Surgery, Hospital General Universitario Gregorio Marañón, Universidad Complutense de Madrid, 28007 Madrid, Spain; cnavarrocuellar@gmail.com

The search for standardized protocols has been a constant concern in Head and Neck Reconstructive Surgery. Nevertheless, the concept of personalization has emerged as a possibility to adjust these protocols to a single individual. This customization of the treatments has been extensively used in medical disciplines for decades and in the last five to ten years, has been incorporated to Surgery.

The aim of this Special Issue, **“New Technologies for Personalized Medicine in Head and Neck Oncologic and Reconstructive Surgery”**, is to offer a particular view of the implications of personalized surgery and customization in the Head and Neck area.

The skill of reconstructive surgery has traditionally been considered to be learning curve dependent. Until recently, overall success in craniofacial reconstruction has relied primarily on the use of 2D imaging modalities, and microsurgical reconstructions of the facial skeleton were performed ‘‘freehand’’ and reconstruction plates were manually adapted during surgery. 

The use of computer-assisted surgery (CAS) and navigation technology in head and neck oncology was first described in 1995 by A. Wagner [1].

Virtual surgical planning (VSP), design, modeling (CAD-/CAM: computer assisted design, computer assisted manufacturing) and surgical navigation techniques have contributed during the last years to simplify and improve the accuracy of this specific type of surgery [2,3,4]. These technologies have gained significant acceptance in oncologic applications. In the area of reconstructive surgery, it provides many benefits, since the surgical precision required to restore facial symmetry, appearance, and function is a complex challenge and the three-dimensional (3D) position is difficult to control, especially in extensive bony defects [5,6,7,8,9,10,11].

Virtual surgical planning and computer-aided design (CAD) allows preplanning of the oncologic resection, flap dimensions, and osteotomies in the bone flap [12]. Computer-aided manufacturing (CAM) cutting guides allow surgeons to accurately perform planned resections and osteotomies, which has improved the precision, accuracy, and reliability of the results of bone resections and reconstructions [4,9,13,14]. Surgical navigation has improved reliability and outcomes by providing real-time feedback to the surgeon [4]. 

VSP began to be used in bone flaps and is now also used in soft tissue flaps for precise localization of the skin perforators of the flap and in ablative virtual surgery to enhance the localization of the soft tissue flaps. In the latter, it is used for precise localization of the skin perforators of the flap and in ablative virtual surgery to establish the reconstructive needs and the most appropriate flap thickness for each reconstruction.

The reconstructive benefits of CAD-CAM implementation include:

1. It enables preoperative visualization of the patient’s individual anatomic features [9,15].
2. It simplifies the osteotomies during tumor ablation in oncologic patients [9].
3. It improves reconstructive accuracy [5,7,8,9].
4. It improves the osteosynthesis of the bone segments for reconstruction [8].
5. It increases the precision of the bone contact surfaces of the flap and the remnant bone, achieving a better aesthetic contour and lower complication rates [5,8].
6. It allows for preoperative visualization of reconstructive limitations and possible complications [9].
7. It increases the possibilities of obtaining clear resection margins, because the surgeon has a 3D visualization of the lesion and an understanding of the future bone defect and the immediate reconstructive plan, thus enhancing the decrease in local recurrence rates [2].
8. It decreases intraoperative time, specifically the decrease in the ischemic time of the microsurgical flap, better reliability in the results and the simplicity of use of this approach [5,8,9,11,14].
9. With surgical navigation, the time needed to identify the perforating vessels can be reduced [4,16,17].
10. Improvement in the predictability of the results, improving patient satisfaction, which means lower total cost (due to shorter surgical time, shorter hospital stay, and lower complication rates) that can potentially offset the technological costs [5,8,9,14].

In recent years, different “In House” navigation systems have been implemented for the placement of dental implants by means of dynamic navigation techniques in oncologic patients. This dynamic navigation technique allows placing the implants with sub-millimeter precision since the apical linear deviation is less than 1 mm and the angular deviation is less than 3°. The result is increased precision and accuracy in implant placement and prosthetic rehabilitation.

On the other hand, the main disadvantages of virtual surgery planning are: (1) increased costs, often due to the need for an external digital laboratory; (2) the surgical delay involved in surgical planning and obtaining the different models and cutting guides, which can delay the beginning of treatment in oncologic patients.

Currently, the introduction of 3D technology and its use in Virtual Planning, Navigation, and Custom-Made prosthesis, have opened several debates.

One of the most important is related to the precision and accuracy of these methods. As the main challenge in the reconstructive surgery of this region is to achieve optimal function and aesthetics, despite its complex three-dimensional (3D) anatomy, it seems that these new technical possibilities are indicated to approach Head and Neck pathology [17].

In this sense, the disadvantage of conventional techniques lies in trying to reconstruct a complex 3D structure by 2D imaging and planning. For complex cases, it can be time-consuming, and unreliable. The use of 2D techniques can negatively influence both the functional and esthetic outcomes [18]. It seems that personalized planning and modeling **optimize aesthetic outcomes and functional rehabilitation** [19].

Another important aspect is related to the **delay of treatment** when we use these techniques. The pretreatment interval is defined as the time from diagnosis to the beginning of treatment and can be influenced by the patient, the health system, and the disease. The expansion of this time can compromise the prognosis, since during this time interval the tumor can multiply and metastasize [20]. Planning and personalized prosthesis manufacturing could increase this period. During the last five years, producers have made a big effort to reduce this interval to no more than two weeks and in selected cases, in-house could potentially reduce the time by 24 h [21].

Another issue is related to the risk of achieving **free margins** in the resection when it is virtually planned. Several studies conclude that cancer patients can be safely treated via primary reconstruction with the help of virtual planning and guided surgery. It could be related to the fact that it is easier to be more aggressive while operating on an image than on an actual body [22]. 

On the other hand, the reduction in operating time is another factor that has several implications related to a better outcome for the patient and reducing overall costs. There is a long-established principle that correlates the acquisition of new technologies with increased costs, but this is not totally true if we take into account the biological costs related to increased operating time and prolonged hospitalization [23,24]. 

Finally, it is important to highlight how the implementation of all these new technologies improves the quality of life of patients. For this reason, it is necessary to carry out quality of life studies that relate the personalized treatment of patients and its results on their quality of life.

In the following years, we expect a **severe economic crisis** that will put some pressure on health systems around the world to reduce costs, and improving the quality of treatments.

In the present Issue, we will analyze the impact of personalization in Craniofacial Surgery. We have invited the most prominent authors who have experience in this field of knowledge, to offer deep insight into the panorama of the use of new technologies.
